# Two different phase-change origins with chemical- and structural-phase-changes in C doped (1.5 wt.%) In_3_Sb_1_Te_2_

**DOI:** 10.1038/srep38663

**Published:** 2016-12-08

**Authors:** Y. M. Lee, S. Y. Lee, T. Sasaki, K. Kim, D. Ahn, M.-C. Jung

**Affiliations:** 1 Beamline Department, Pohang Accelerator Laboratory (PAL), Pohang, 37673, Republic of Korea; 2 Biology Resources Section, Research Support Division, Okinawa Institute of Science and Technology Graduate University, Okinawa, 904-0495, Japan; 3 AE Center, Samsung Advanced Institute of Technology, P. O. Box 111, Suwon, 440-600, Korea; 4 Laboratroy for Organic Electronics, Graduate School of Materials Science, Nara Institute of Science and Technology, Nara, 630-0192, Japan

## Abstract

We fabricated C-doped (1.5 wt.%) In_3_Sb_1_Te_2_ (CIST) thin films with amorphous phase (*a*-CIST) using a sputter method. Two electrical-phase-changes at 250 and 275 °C were observed in the sheet resistance measurement. In order to understand the origin of these electrical-phase-changes, all samples were characterized by XRD, TEM, and HRXPS with synchrotron radiation. In *a*-CIST, only weak Sb-C bonding was observed. In the first electrical-phase-change at 250 °C, strong Sb-C bonding occurred without an accompanying structural/phase change (still amorphous). On the other hand, the second electrical-phase-change at 275 °C was due to the structural/phase change from amorphous to crystalline without a chemical state change.

Phase-change materials have potential applications in non-volatile random access memory devices (PRAM), where they may eventually replace Si-based chips^1^,^2^. The utility of these materials stems from extreme changes in both optical reflectivity and electrical resistance during the amorphous-to-crystalline phase change at low temperature^3^. Most researchers have studied Ge_2_Sb_2_Te_5_ ternary alloys (GST) which are widely used in memory devices because of their proper phase-change temperature of 180 °C and their high phase-change speed^4^. To improve properties of phase-change materials, so as to achieve more stable amorphous phases, faster phase transitions, higher integration, etc., recently studies have employed doping in the hope of finding new phase-change materials^5^,^6^,^7^,^8^,^9^. In_3_Sb_1_Te_2_ (IST) is a good candidate for phase-change, random access memory materials because it undergoes multiple phase changes^9^. Previously, we determined that the phase-change mechanism of IST originated from chemical phase separation during the phase-change and we assumed that it is non-reversible because of Sb structural instability^10^. However, it is important to continue investigating the multi-phase-change property of IST because of its potential to dramatically increase the integration of memory devices. To overcome the structural instability of the IST phase-change, doping studies are a reasonable starting point. Recently, Kim *et al*. reported that a carbon-doped IST displayed higher retention than an IST ternary alloy^11^. In case of device feature, this result is very promising in PRAM commercial product. However, the phase-change mechanism was not fully understood in physical and chemical terms because they used only transmission electron microscopy (TEM) to investigate the phase-change trend^11^.

In this study, we fabricated a C-doped (1.5 wt.%) In_3_Sb_1_Te_2_ (CIST) thin film in an amorphous phase (*a*-CIST) using a sputter method. In order to understand the origin of electrical-phase-changes, all samples were characterized with x-ray diffraction (XRD) and high-resolution x-ray photoelectron spectroscopy (HRXPS) with synchrotron radiation.

## Results and Discussion

**Figure 1:**
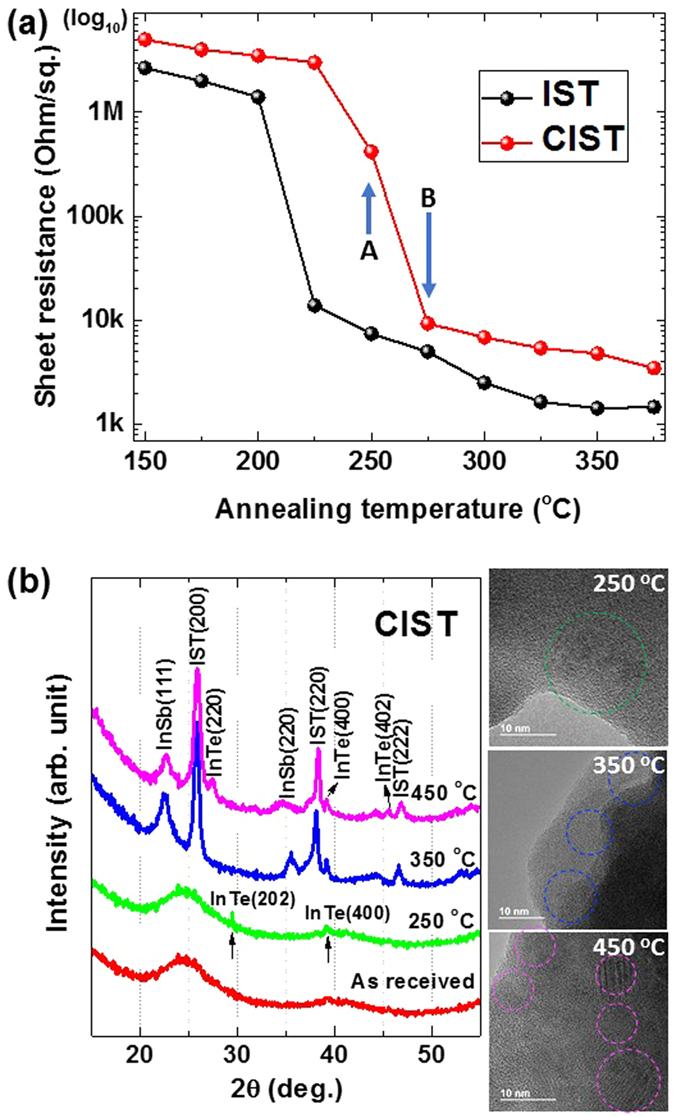
(**a**) Sheet resistance and (**b**) structural information using HRXRD and TEM during the phase-change. At ~350 °C, the phase-change of *a*-CIST was indicated by a change of resistance and crystallinity.

To document the resistance change, we measured the sheet resistance while increasing the temperature from 150 °C to 400 °C (Fig. 1a). Around 225 °C in the pure IST sample, we observed a large change in sheet resistance. However, the sheet resistance of CIST is changed at 250 °C (A in the Fig. 1a). The sheet resistance also changed again at 275 °C (B in the Fig. 1a). This confirmed that the carbon doping effect in IST was due to the increased phase-change temperature. This result is similar to that of nitrogen doping in GeTe and GST^5^,^12^,^13^.

To investigate the physical origin of these sheet resistance changes in more detail, the structures of all of the samples annealed at elevating temperature were characterized by XRD and TEM (Fig. 1b). When the sample was annealed to around 250 °C, broad peaks by a typical amorphous phase (*a*-CIST) are observed in the XRD patterns except two very low-intensity peaks at 29° and 39° 2*θ* that could be indexed as (202) and (400) crystallographic plane of InTe phase^14^. Additionally, this crystalline phase was confirmed by observation of the lattice fringes in the TEM image (Fig. 1b). From the diffraction peaks in the XRD patterns at annealing temperatures at 350 °C and 450 °C, we confirmed that most of *a*-CIST phase transformed into crystalline phases which correspond to cubic InSb, cubic In_3_SbTe_2_ (*c*-IST), and tetragonal InTe phase as shown in Fig. 1(b), respectively. As the annealing temperature increased from 350 °C to 450 °C, the XRD peaks of *c*-IST (200), (220), and (222) moved to the higher 2*θ* angle direction, indicating that the plane spacing of the sample is shorter. However, it was reported that IST phase is metastable under 420 °C in the phase diagram of IST^15^,^16^,^17^. Moreover, Eun Tae Kim *et al*. reported that the IST peak appears at 400 °C and the peak intensity is maximum at 450 °C^9^. According to PDF #17-0849 (Fm-3m, *a* = 6.1263 Å), the d value of (200) diffraction peak of IST is 3.05 Å. In the present work, the interplanar (d) value of (200) diffraction peak of *c*-IST at 350 °C and 450 °C are 3.131 Å and 3.124 Å, respectively. Therefore, the peak shifts in the XRD patterns were attributed to phase transformation of *c*-IST from thermodynamically metastable state to stable state.

From the sheet resistance measurement, we assumed that CIST underwent a structural phase change at 250 °C. However, from the XRD results it is apparent that this was an electrical phase-change without an associated structural phase-change. This suggests that the first electrical phase-change may have resulted from a chemical phase-change (Fig. 1a)^18^. XRD results confirmed that the second change of sheet resistance (Fig. 1a) was due to a structural phase-change.

**Figure 2:**
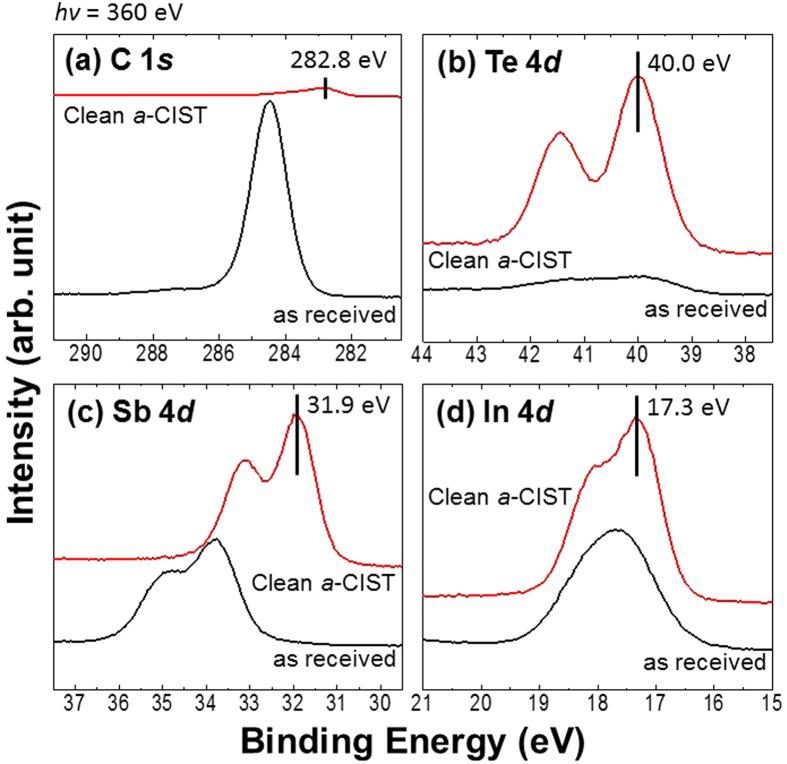
(**a**) C 1*s*, (**b**) Te 4*d*, (**c**) Sb 4*d*, and (**d**) In 4*d* core-level spectra before and after mild Ne^+^ sputtering. We could not observe the O 1*s* core-level after sputtering.

To understand these independent electrical and structural phase-changes in CIST, we measured HRXPS with synchrotron radiation. To remove surface oxide, samples were mildly sputtered under Ne^+ ^18,19,20,21. This resulted in a clear improvement of peaks in C 1*s*, Te 4*d*, Sb 4*d*, and In 4*d* core-levels (Fig. 2). Peak intensity of O 1*s* core-level that originated from the surface oxide completely disappeared. After sputtering, peak binding energies of Te, Sb, and In 4*d* core-levels were 40.0, 31.9, and 17.3 eV, respectively. These binding energies are different with pure IST^10^. The chemical shift of Sb 4*d* core-levels between pure and C-doped IST was 0.3 eV. In the case of C 1*s* core-level, we observed a binding energy of 284.5 eV before sputtering. However, this peak completely disappeared and a new peak with a binding energy around 282.8 eV was observed after sputtering. This means that the binding energy peak at 284.5 eV originated from the surface oxide of C-doped IST. Normally, C-C bonding is observed at 284.5~284.8 eV^22^,^23^. Also if the carbon is cationic, its binding energy will be shifted higher (e.g., C-O, ~286 eV and CF_2_, ~292 eV) than that of C-C bonding^22^. However, a chemical state at a lower binding energy than C-C indicates a metal carbide species^22^. On the other hand, the binding energy of Sb 4*d* core-level shifted to a higher value (31.9 eV) than the Sb of pure IST. This implies a cationic role. Finally, the doped carbon in *a*-CIST is bonded with Sb, creating an Sb-C metal carbide. This is completely different than C- or N-doped GST^12^,^20^,^24^. In the latter case, in Fe- or Mn-doped IST, these metals bonded only with In atom in the amorphous phase^18^,^21^. Here, the *a*-CIST has the chemical state of an Sb-C metal carbide.

**Figure 3:**
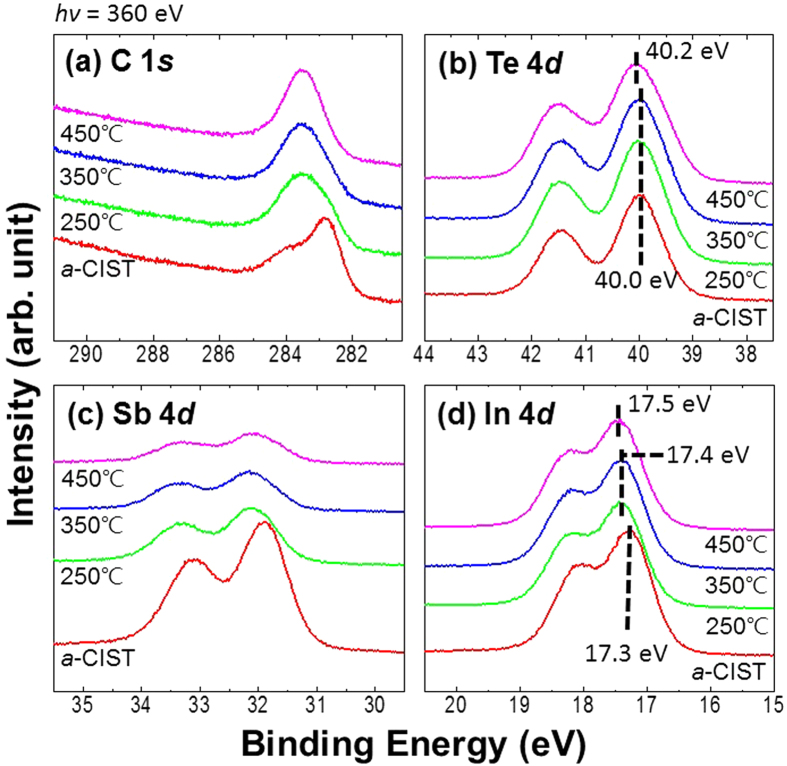
(**a**) C 1*s*, (**b**) Te 4*d*, (**c**) Sb 4*d*, and (**d**) In 4*d* core-level spectra after annealing at each temperature. The C 1*s* and Sb 4*d* core-levels changed with different chemical states and decreasing peak intensity, respectively.

In order to see the change of chemical states during both electrical and structural phase-changes, we measured HRXPS from 250 to 450 °C (Fig. 3). At 250 °C, significantly, a new chemical state in C 1*s* core-level appeared at 283.6 eV and maintained its peak intensity and shape in spite of increasing temperature (Fig. 3a). In the case of Te 4*d* core-level (Fig. 3b), the binding energy at 40.0 eV did not change except in the 450 °C sample. The peak in the 450 °C sample was observed at 40.2 eV. A dramatic change was seen in Sb 4*d* core-level spectra (Fig. 3c). At 250 °C, peak intensity decreased dramatically by half and then continued to decrease further with increasing temperature. This means that Sb is depleted from the surface. In a device, this is very important, because Sb will diffuse to the top electrode in the isolated cell space of the device. Sb diffusion will create a type of thin film in the cell and will change the electrical properties of the device. If C-doped IST-based phase-change random access memory is fabricated, Sb stoichiometry should be considered in order to avoid this diffusion. The peak position also changed from 31.9 to 32.1 eV. This means that the Sb-C bond developed more metallic properties. We believe that this new, stronger Sb-C bond created at 250 °C is induced during the first electrical phase-change without a structural phase-change. In the case of In 4*d* core-level spectra (Fig. 3d), we observed changes of binding energy from 17.3 at *a*-CIST to 17.4 eV at 250 and 350 °C. At 450 °C the binding energy was 17.5 eV. Binding energies of In 17.5 eV and Te 40.2 eV are matched with InTe^10^,^25^, and this result is consistent with XRD data.

**Figure 4:**
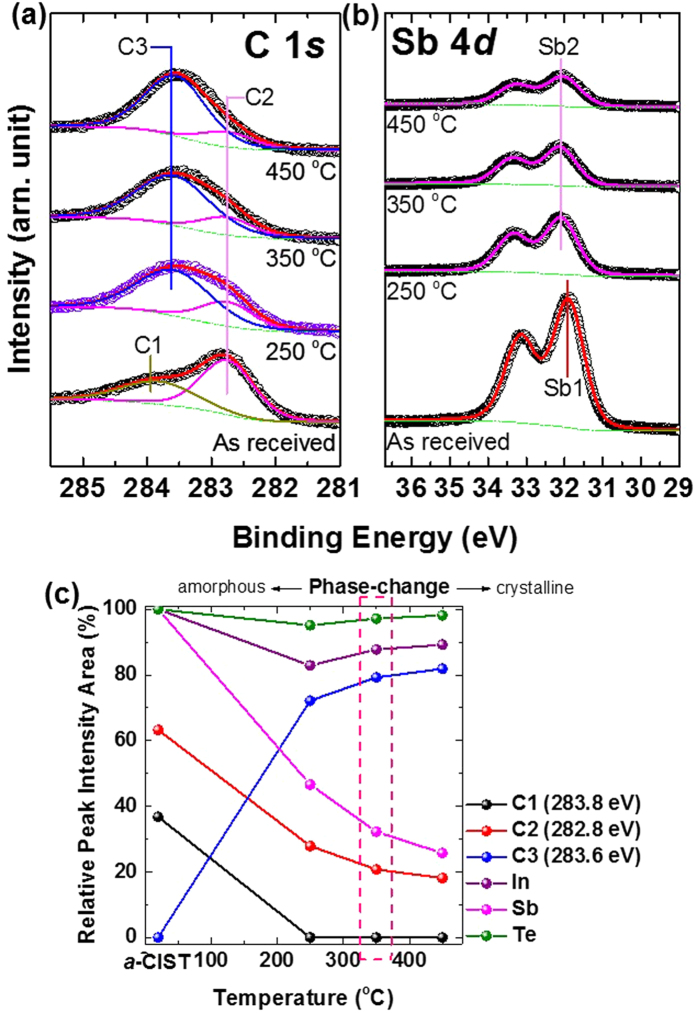
Curve-fitting of (**a**) C 1*s* and (**b**) Sb 4*d* after annealing at each temperature. (**c**) The relative peak intensity area with the function of each element. The intensity of Sb 4*d* core-level decreased >60%.

In order to analyze the spectra of C 1*s* and Sb 4*d* core-levels in more detail, we performed curve-fitting (Fig. 4a,b) using Doniach-Sŭnjić curves, convoluted with a Gaussian distribution function, considering instrumental broadening^26^. Background noise due to inelastic scattering was subtracted by the Shirley (integral) method^27^. In the curve-fitting of C 1*s* core-level, we found three chemical states, C1, C2, and C3 with binding energies of 283.8, 282.8, and 283.6 eV, respectively. The C2 chemical state (weak Sb-C bonding) is observed in all samples with different peak intensities. As the temperature increased to 250 °C, the C1 chemical state (a kind of C-C bonding) disappeared completely and the C3 chemical state (strong Sb-C bonding) appeared. On the other hand, in Sb 4*d* curve fitting, the chemical state of Sb1 was observed only in *a*-CIST and after increasing the temperature we observed only the Sb2 chemical state. Binding energies of Sb1 and Sb2 were 31.9 and 32.1 eV, respectively. Sb1(Sb-C bonding) and Sb2 (relatively strong Sb-C bonding than Sb1) are bonded with C2 and C3, respectively. The chemical state of Sb2 is not changed in the 2nd phase-change. If we have the new chemical state of C3 after 1st and 2nd phase-changes, normally we should observe a chemical state of Sb3 (for new cation element). However, we could not observe. It means that the chemical state of C is only changed without a new chemical state of Sb. This means that at the 2nd phase-change the chemical environment of Sb is not changed but the next nearest neighbor (the C element) is changed. In this case, we could not mention the stoichiometry. That reason was why we made the relative labels of “Strong Sb-C bonding” and “Weak Sb-C bonding”. Also, the peak intensity of Sb 4*d* core-level decreased more than 60% as temperature increased (Fig. 4c).

Interestingly, the chemical states of CIST at 250 °C had already changed without a structural phase-change. This means that the first electrical phase-change of CIST is due to the new, strong Sb-C chemical bonding. Then structural ordering followed at 350 °C. At that time, the second electrical phase-change occurred without a chemical state change. Finally, we found that the first and second electrical phase-changes of CIST originated from chemical phase-changes (with strong Sb-C bonding) and structural phase-change (from amorphous to crystalline), respectively.

## Conclusions

We fabricated *a*-CIST and performed post-annealing experiments to understand phase-change mechanisms in CIST. All prepared samples with different annealing temperatures were characterized in terms of sheet resistance, XRD, TEM, and HRXPS. The formation of Sb-C bonding, a kind of metal carbide, in *a*-CIST is very different than C- or N-doped GeTe or GST. We found that CIST underwent two electrical phase-changes and the origins of first and second electrical phase-changes in CIST are a chemical phase-change (strong Sb-C bonding) and a structural phase-change (from amorphous to crystalline).

## Methods

### Sample preparation

C-doped *a*-IST thin films (100 nm) were deposited onto Si(001) substrates in an Ar atmosphere using reactive sputtering with a single CIST target at room temperature. To remove the oxide layers formed on the surface of the thin film by air exposure, the C-doped *a*-IST was etched with Ne^+^ (99.999%) ion sputtering for 1 h with an ion beam energy of 1 kV under a pressure of 1.0 × 10^−6^ *Torr* to remove any surface oxide^10^,^21^. We used the resistivity heating method and a K-type thermocouple on a Si substrate to apply heating and measure the temperature, respectively^10^,^28^.

### Characterization using TEM, HRXRD and HRXPS with synchrotron radiation

For TEM measurements, we used an ARM200F (JEOL) which employs atomic resolution TEM with the S-TEM Cs corrector. All samples were scraped on the surface to get sample shavings. Shavings were mounted on TEM grids. In order to confirm structural phases of samples, we performed XRD at the 9C beamline of the Pohang Light Source II (PLS-II) in South Korea. X-ray energy of 8.9 keV (λ = 1.3932 Å) was selected by a double crystal Si(111) monochromator, and XRD data were obtained from 15°–55° with a standard theta-two-theta scan. HRXPS spectra were obtained using synchrotron radiation at the 10D beamline of the Pohang Light Source II. Photon energy was varied from 360 eV (for C 1*s*, Te 4*d*, Sb 4*d*, and In 4*d* core-level) to 660 eV (for O 1*s* and Sb 3*d* core-level) to obtain high-quality XPS spectra. Photoelectron signals were recorded with a PHOIBOS 150 electron energy analyzer equipped with a two-dimensional charge-coupled detector (2D CCD) (Specs GmbH), collecting photoelectrons normal to the surface. The binding energy scale was calibrated with the Au 4*f* core-level peak at 84.0 eV^29^. The base pressure of the main chamber was maintained below 1.2 × 10^–10^ *Torr*.

## Additional Information

**How to cite this article**: Lee, Y. M. *et al*. Two different phase-change origins with chemical- and structural-phase-changes in C doped (1.5 wt.%) In_3_Sb_1_Te_2_. *Sci. Rep.* **6**, 38663; doi: 10.1038/srep38663 (2016).

**Publisher's note:** Springer Nature remains neutral with regard to jurisdictional claims in published maps and institutional affiliations.
